# Gold Nanoparticle-Enhanced Production of Reactive Oxygen Species for Radiotherapy and Phototherapy

**DOI:** 10.3390/nano15040317

**Published:** 2025-02-19

**Authors:** Viet-Khang Nguyen, Shiao-Wen Tsai, I-Chun Cho, Tsi-Chian Chao, Ing-Tsung Hsiao, Hsiao-Chieh Huang, Jiunn-Woei Liaw

**Affiliations:** 1 Department of Mechanical Engineering, Chang Gung University, Taoyuan City 33302, Taiwan; d000018133@cgu.edu.tw; 2 Department of Biomedical Engineering, Chang Gung University, Taoyuan City 33302, Taiwan; d000000608@cgu.edu.tw; 3 Radiation Research Core Laboratory, Chang Gung Memorial Hospital, Taoyuan City 333034, Taiwan; d000018305@cgu.edu.tw (I.-C.C.); chaot@gap.cgu.edu.tw (T.-C.C.); 4 Research Center for Radiation Medicine, Chang Gung University, Taoyuan City 33302, Taiwan; 5 Department of Medical Imaging and Radiological Science, Chang Gung University, Taoyuan City 33302, Taiwan; ihsiao@mail.cgu.edu.tw; 6 Proton and Radiation Therapy Center, Chang Gung Memorial Hospital, Taoyuan City 333034, Taiwan; hsiaochieh@cgmh.org.tw; 7 Department of Mechanical Engineering, Ming Chi University of Technology, New Taipei City 24301, Taiwan

**Keywords:** gold nanoparticles, reactive oxygen species (ROS), radiotherapy, proton beams, organelle damage, secondary electrons

## Abstract

Gold nanoparticles (GNPs) have gained significant attention as multifunctional agents in biomedical applications, particularly for enhancing radiotherapy. Their advantages, including low toxicity, high biocompatibility, and excellent conductivity, make them promising candidates for improving treatment outcomes across various radiation sources, such as femtosecond lasers, X-rays, Cs-137, and proton beams. However, a deeper understanding of their precise mechanisms in radiotherapy is essential for maximizing their therapeutic potential. This review explores the role of GNPs in enhancing reactive oxygen species (ROS) generation through plasmon-induced hot electrons or radiation-induced secondary electrons, leading to cellular damage in organelles such as mitochondria and the cytoskeleton. This additional pathway enhances radiotherapy efficacy, offering new therapeutic possibilities. Furthermore, we discuss emerging trends and future perspectives, highlighting innovative strategies for integrating GNPs into radiotherapy. This comprehensive review provides insights into the mechanisms, applications, and potential clinical impact of GNPs in cancer treatment.

## 1. Introduction

**Figure 1:**
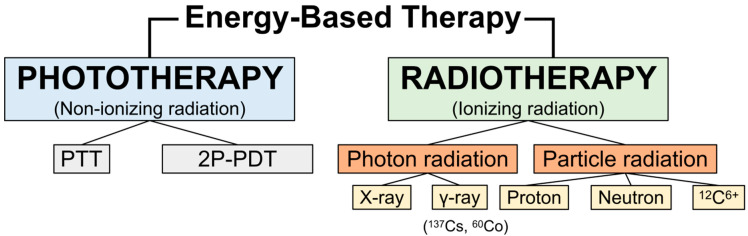
Energy-based therapies, including phototherapy (non-ionizing radiation) and radiotherapy (ionizing radiation), can have their efficacy enhanced by GNPs.

Cancer is a complex and devastating group of diseases characterized by the uncontrolled proliferation of abnormal cells affecting various parts of the human body. According to the International Agency for Research on Cancer (IARC) (https://gco.iarc.fr/, accessed on 5 November 2024), the incidence and mortality of all cancers are expected to experience a substantial surge from 2020 to 2040, with estimated new cases increasing from 19.3 to 30.2 million and estimated deaths rising from 9.96 to 16.3 million. Significantly, radiation therapy, also known as radiotherapy, plays a crucial and well-established role in cancer treatment by employing ionizing radiation to create ions within tissues, strip electrons from atoms and molecules, and potentially lead to cell death or gene alterations that inhibit cell growth, all while selectively targeting and destroying malignant cells and minimizing harm to healthy tissues [1,2]. Progress in the development of radiotherapy has centered on sophisticated technology and the provision of advanced tools for precise radiation delivery. Approximately 31% of all cancer patients receive radiation therapy as part of their initial treatment, and over 50% require it at some point during their disease [3]. This therapy employs high-energy radiation to induce DNA breaks in cancer cells, combined with excessive reactive oxygen species (ROS) production, endoplasmic reticulum (ER) stress, and mitochondrial dysfunction, ultimately triggering apoptosis and inhibiting cancer cell proliferation [4]. Different types of ionizing radiation can be grouped into either photon radiation, such as X-rays and gamma rays, or particle radiation, which includes electrons, protons, neutrons, carbon ions, alpha particles, and beta particles, and the level of energy determines the depth of tissue penetration [5,6,7,8]. In addition to ionizing radiation therapies, photodynamic therapy (PDT) and photothermal therapy (PTT), a non-ionizing technique, uses visible or near-infrared light to activate photosensitizers that generate ROS or heat, offering a complementary approach for targeted cancer treatment (Figure 1) [9,10]. In addition, femtosecond pulsed lasers can induce two-photon excitation (TPE), enhancing the efficacy of PDT [10]. Understanding the multifaceted nature of radiation therapy, which involves factors such as radiation type, dosing regimen, delivery technique, and the biological characteristics of the tumor and surrounding tissues, is essential for developing novel strategies aimed at improving radiotherapeutic outcomes [1].

Despite its efficacy, radiotherapy faces limitations, including tumor resistance to radiation and the risk of harming healthy tissue. Radioresistance in cancer is primarily driven by various mechanisms such as aberrant DNA damage repair, gene mutations, dysregulated signaling pathways, alterations in cell cycle, tumor microenvironment and metabolic heterogeneity, autophagy, and ferroptosis, requiring different molecular interventions depending on tumor subtypes [11,12,13,14,15,16,17]. Acute side effects, additionally occurring during or shortly after treatment, mainly affect rapidly dividing tissues, while late effects typically impact more slowly proliferating tissues, often resulting in conditions such as fibrosis, vascular injury, hormonal imbalance, and second malignancies [18,19,20,21,22,23,24,25]. As tumor cells acquire radioresistance, the need for increased radiation doses in treatment may lead to potential harm or even fatality to normal cells and tissues. Several strategies have been suggested to achieve a balance between treatment effectiveness and minimizing adverse effects. These include enhancing radiosensitivity and reversing radioresistance in tumor tissue, restricting the delivery of radiation dose to the tumor site, and radioprotecting adjacent tissue [26]. Radiation sensitization is the process of increasing the susceptibility of tumor tissues to radiation-induced damage, and radiosensitizers are agents that boost the effects of radiotherapy. Over the past decade, high atomic number (high-Z) metal-based nanoparticles, including silver (_47_Ag), gadolinium (_64_Gd), hafnium (_72_Hf), platinum (_78_Pt), gold (_79_Au), and bismuth (_83_Bi), have been investigated for their potential in enhancing the precision of radiotherapy by selectively scattering with high-energy radiation to target tumor cells [9,27,28,29,30,31,32,33,34,35]. Gold nanoparticles (GNPs), in particular, have emerged as a promising tool for cancer diagnosis and treatment due to their chemical inertness, strong photoelectric interaction, high surface-to-volume ratio and biocompatibility, tumor-targeting capabilities via the enhanced permeability and retention (EPR) effect, and versatility in disease diagnosis and bioimaging [26,36,37,38,39,40,41,42,43]. Depending on various parameters, including particle size and morphology, surface characteristics, concentration, as well as biodistribution and specific localization, the effectiveness of GNPs in radiotherapy can vary widely [43,44,45,46]. Smaller GNPs produce more low-energy secondary electrons, enhancing radiation dose deposition in the surrounding medium. In contrast, larger GNPs trap more ionizing events within their structure, reducing the energy available for interactions with nearby biological targets [47]. Simulations and experimental studies indicate that nanoparticles around 2–3 nm are more effective in inducing cell damage than larger ones, as secondary electrons in bigger particles lose energy internally before escaping to interact with surrounding tissues [35,48]. Although shapes such as nanorods and nanostars have special advantages, nanospheres remain the most widely studied and applied in radiotherapy due to their stability and convenience of size optimization. These nonspherical GNPs enhance localized electric field effects and SPR due to their anisotropic geometry, potentially amplifying electron ejection and ROS generation [49,50]. Positively charged nanoparticles enhance cellular uptake by interacting with negatively charged lipid membranes and potentially selectively target cancer cells due to their larger, more negatively charged glycocalyx (a protective layer of glycoproteins and glycosaminoglycans on the cell surface) [51]. By modifying surface charge, stability, and functionalization, GNP coatings can fine-tune interactions with specific biological targets. GNP targeting in tumors involves passive targeting and active targeting, which utilize leaky vasculature and high endocytic uptake, and functionalized nanoparticles binding to tumor-specific receptors, respectively [52,53,54]. When the immune system detects foreign particles, opsonins (defensive proteins) attach to them, signaling for their removal. In passive targeting, poly(ethylene glycol) (PEG)-coated GNPs prevent opsonin adsorption, thereby reducing immune recognition, enhancing circulation time, and tumor accumulation through the EPR effect. In contrast, active targeting requires conjugating specific molecules such as antibodies, peptides, folates, or aptamers to GNPs to interact with tumor cells, avoiding reliance on passive uptake via the EPR effect [51].

**Figure 2:**
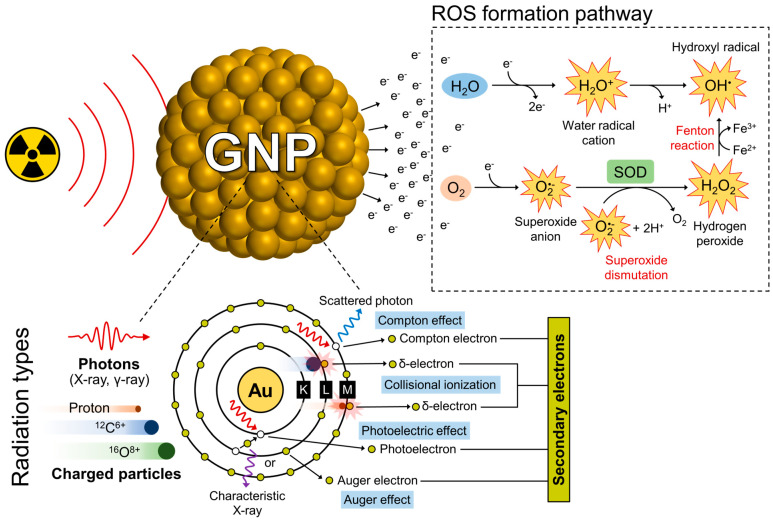
Formation of ROS via secondary electrons from GNPs under radiation.

Radiosensitization by GNPs involves not only physical dose enhancement through strong photoelectric absorption but also subsequent chemical and biological processes. Under radiation, GNPs generate excess ROS via secondary electron interactions, leading to oxidative stress, DNA damage, lipid peroxidation, and apoptosis, which collectively enhance radiotherapeutic effectiveness [9,32,55,56,57]. High-energy photons or charged particles interact with GNPs, leading to the emission of various secondary electrons, including photoelectrons, Auger electrons, Compton electrons, and δ-electrons (Figure 2). The therapeutic efficacy of GNPs in radiotherapy depends significantly on the type of radiation used. Low-energy X-rays (<100 keV) enhance localized dose deposition through strong photoelectric absorption, leading to increased ROS production. In contrast, high-energy X-rays and gamma rays (>1 MeV) primarily induce Compton scattering, which results in a more diffused energy deposition pattern with potentially lower local ROS generation [32,58,59]. Proton therapy, although primarily reliant on direct ionization, can also benefit from GNPs due to secondary electron emission and localized proton energy deposition in the Bragg peak region, potentially improving tumor control while sparing surrounding healthy tissues [55,59,60,61,62,63]. When exposed to charged particles such as protons or heavy ions, additional δ-electrons are produced due to inelastic collisions with atomic electrons [64]. These high-energy secondary electrons subsequently interact with surrounding water molecules, inducing ionization and radiolysis, generating primary radicals such as water radical cation (H_2_O^+^) and hydrated electrons. These radicals then undergo redox reactions to form ROS, including hydroxyl radicals (OH^•^), superoxide anions (O_2_^•−^), and hydrogen peroxide (H_2_O_2_), which contribute to oxidative stress and enhance cancer cell damage (Figure 2) [58,65]. In addition, femtosecond laser irradiation of GNPs enhances ROS production through TPE and surface plasmon resonance (SPR) [10]. Consequently, SPR may generate hot electrons, further inducing ROS in the surrounding medium. However, the SPR effect in GNPs occurs only in the visible to near-infrared region.

Understanding the intricate interplay between GNPs and radiotherapy is essential for enhancing therapeutic outcomes and enabling personalized cancer treatment strategies. This review explores the radiosensitizing mechanisms of GNPs, emphasizing their interactions with different radiation sources and their role in treatment. Focusing on radiosensitization via ROS across various radiation types, it offers a comparative analysis of their effectiveness in different radiotherapy modalities, distinguishing it from recent reviews that address general radioenhancement mechanisms, clinical translation, or specific therapy combinations [61,66,67,68].

## 2. GNPs as Radiosensitizers Under Various (Non)-Ionizing Radiations

### 2.1. GNPs in Two-Photon Photodynamic Therapy

**Figure 3:**
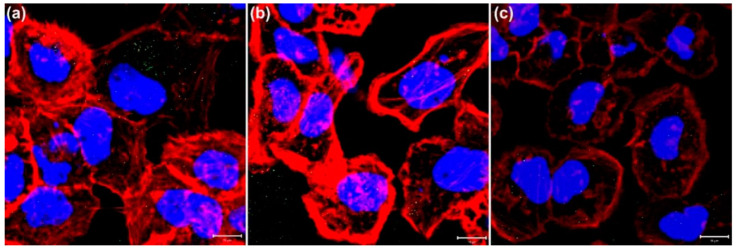
Confocal fluorescence images of the cellular cytoskeleton illustrating the inhibitory effect of NAC on ROS in GNP-treated A431 cells: (**a**) 5 mM NAC; (**b**) 10 mM NAC; (**c**) no NAC. F-actin (red) and nuclei (blue) were stained with Texas Red-X phalloidin and Hoechst 33342, respectively [10]. Scale bar: 10 µm.

Two-photon photodynamic therapy (2P-PDT) in medical applications involves using a femtosecond pulsed laser to induce the absorption of two near-infrared red (NIR) photons simultaneously for photosensitizer activation, exploiting two-photon absorption and the biological transparency window of NIR light [69]. This approach, for example, with femtosecond NIR lasers at around 800 nm, demonstrates superior efficiency in generating ROS compared with continuous-wave lasers, emphasizing its potential for precise and effective therapeutic interventions [70,71]. Under controlled circumstances, ROS plays a crucial role in biological systems by sustaining a stable redox state. However, excessive ROS generation, facilitated by hot electrons emitted from GNPs through TPE and SPR, leads to cellular organelle damage, ultimately inducing blebbing, apoptosis, and necrosis [9,10,72,73,74,75]. Recently, the presence of GNPs was found to intensify ROS expression in cells exposed to a TPE, leading to elevated cytoskeletal disruption and apoptosis as the number of femtosecond laser shots increases due to 2P-PDT. Furthermore, inhibiting ROS formation with the antioxidant *N*-acetyl-L-cysteine (NAC) during treatment with GNPs not only significantly reduces ROS generation but also mitigates cytoskeletal damage compared with cells treated with GNPs alone (Figure 3) [10]. Although the SPR of GNPs can facilitate the generation of ROS when irradiated by a low-power TPE, future therapeutic uses might benefit from differently shaped GNPs, such as surfactant-free protein-coated gold nanorods, where the efficiency of hot-electron generation is higher [76].

### 2.2. GNPs in X-Ray Radiotherapy

**Figure 4:**
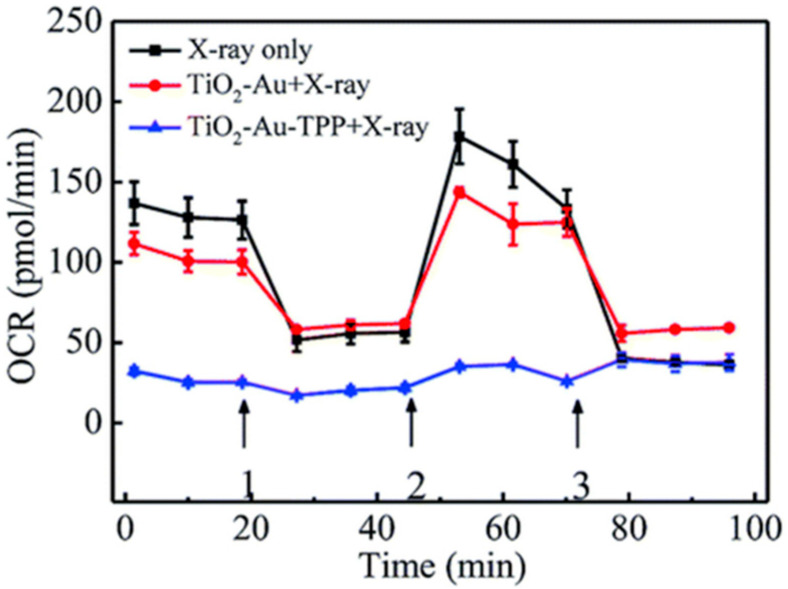
Mitochondrial oxygen consumption rate was measured in real-time using XF24 cell culture plates for cells treated under different conditions [81].

The primary source of X-rays in healthcare stems from Bremsstrahlung interactions, where an electron beam colliding with a high-Z target produces a diverse spectrum of X-ray energies across various angles, resembling the light emitted by an incandescent bulb [77]. Radiotherapy primarily utilizes high-energy X-rays in the megavoltage (MV) range, enabling the treatment of internal tumors while minimizing impact on the skin. Previous studies demonstrated that GNP-induced DNA damage in an MV beam is influenced by treatment factors affecting the proportion of low-energy photons, with a notable increase observed in damage during flattening filter-free delivery [78,79,80]. The combination of chemotherapeutics, such as docetaxel, with GNP in radiotherapy has demonstrated a significant enhancement in therapeutic effects on cancer cells. This is evidenced by increased DNA double-strand breaks and a substantial reduction in tumor growth, ultimately resulting in the complete survival of mice [33]. Li et al. showed that titanium dioxide-gold-triphenylphosphine (TiO_2_-Au-TPP) nanosensitizers, when combined with X-ray irradiation, significantly reduced mitochondrial oxygen consumption in MCF-7 cells, leading to mitochondrial dysfunction and enhanced cell apoptosis (Figure 4) [81]. This effect was associated with increased mitochondrial ROS production and a reduction in membrane potential, suggesting that TiO_2_-Au-TPP could improve radiotherapy outcomes by targeting cellular energy metabolism [81]. Recently, tailored GNPs with functional modifications have been designed to enhance responsiveness to megavoltage radiation (Table 1) [33,82,83,84,85,86,87,88,89,90,91,92,93,94,95,96,97,98].

### 2.3. GNPs in γ-Ray Radiotherapy

**Figure 5:**
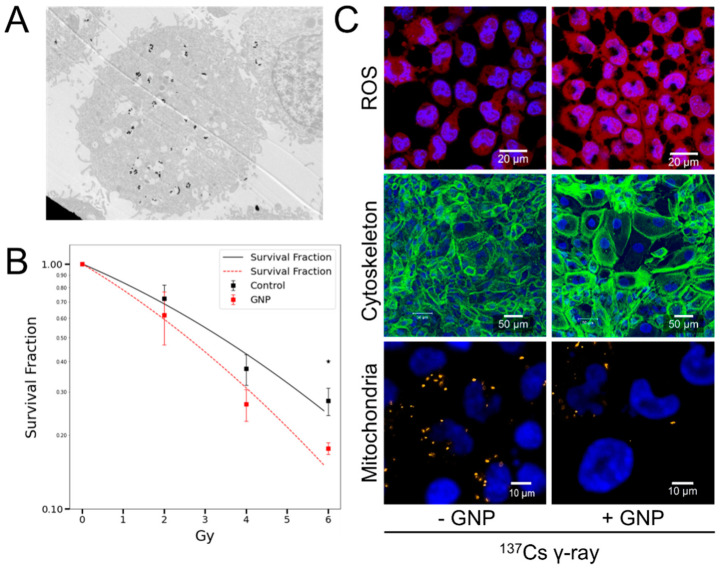
Radiosensitization of GNPs in A431 cells by 137Cs γ-ray irradiation. (**A**) TEM image showing the localization of GNPs within the cells via endocytosis; (**B**) enhanced cell death induced by GNPs; (**C**) generation of ROS and damage to cellular organelles (cytoskeleton and mitochondria) [32]. ★: *p*-value < 0.02.

In contrast to X-rays, which are also widely used in medical imaging and radiation therapy, γ-rays from radioactive sources such as cobalt-60 (^60^Co), cesium-137 (^137^Cs), iridium-192 (^192^Ir), and technetium-99m (^99m^Tc), exhibit higher energy levels. These isotopes emit high-energy γ-rays that penetrate tissues deeply, allowing for precise and effective treatment of tumors [5,32,99,100,101,102,103]. Several studies have been conducted to design GNPs with diverse morphologies and surface modifications to enhance cellular uptake, serving as radiosensitizers for ^137^Cs and ^60^Co γ-ray applications (Table 2) [60,84,104,105,106,107,108,109]. Tsai et al. demonstrated that non-functionalized GNPs enhance the tumoricidal efficacy of ^137^Cs γ-ray radiotherapy by increasing ROS production, leading to greater damage to tumor cell organelles (cytoskeleton and mitochondria) and improved radiosensitization, especially for radioresistant tumors (Figure 5) [32]. PEG-coated GNPs, particularly those with sizes of 12.1 and 27.3 nm, exhibit enhanced sensitization effects, effective tumor accumulation, and a significant reduction in cancer cell survival and tumor volume after radiation while causing liver damage and gold accumulation without adverse effects on the spleen and kidneys [60]. Cyclic RGD-coated ultrasmall GNPs of 1.8 nm exhibited significant uptake by hepatocytes, particularly accumulating inside endosomes within the cytoplasm of ALTS1C1 cells [104]. Tiopronin-covered GNPs of 2 and 6 nm sizes demonstrated both cytoplasmic and nuclear localization in MCF-7 breast cancer cells, maintaining monodispersity within the cell, while larger particles (10 and 16 nm) were restricted to the cytoplasm, suggesting a size-dependent correlation with the nuclear pore complex’s permeability limit [110].

### 2.4. GNPs in Particle Therapy

Energy deposition during X-ray irradiation occurs continuously along the beam path, whereas charged particles, such as proton (^1^H^+^), carbon (^12^C^6+^), oxygen (^16^O^8+^) and helium (^4^He^2+^) ion irradiation, exhibit a distinct dose deposition pattern known as the Bragg peak [111]. The majority of their energy is concentrated at the end of their trajectory, resulting in minimal exit dose and reduced dose deposition along the beam path [112]. This unique characteristic of charged particles allows for sparing healthy tissue and enables more precise targeting of energy within tumor cells. Consequently, particle therapy offers opportunities for focused dose escalation, reducing toxicities and the risk of secondary tumors associated with radiation exposure to healthy tissues [113]. Proton therapy is particularly advantageous for treating tumors near sensitive organs and in specific patient groups due to its superior targeting capabilities [114]. The high-energy proton beams used in clinical practice exhibit a unique characteristic of dropping rapidly in energy at their range’s end, leading to increased ionization density and higher linear energy transfer (LET) [115]. This elevated LET results in more complex and unrepairable biological damage, contributing to an increased relative biological effectiveness (RBE) in the distal region of the spread-out Bragg peak (SOBP) [9,61,116]. Despite the initially anticipated limited efficacy of GNP radiosensitizers in proton therapy due to weak ionization interactions, experimental findings contradicted this expectation, revealing significant enhancements in both in vitro and in vivo studies. The observed radiosensitization was attributed to the increased production of ROS through radiolysis induced by GNPs, suggesting a promising combination of proton therapy’s healthy tissue sparing and GNP’s enhanced RBE for potentially improved patient outcomes [55,59,62,117].

**Figure 6:**
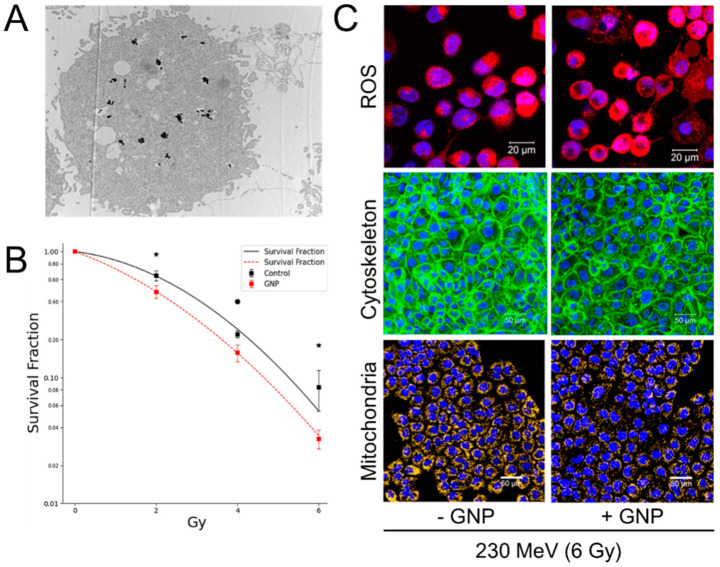
Radiosensitization of GNPs in A431 cells by proton beam therapy. (**A**) TEM image showing the localization of GNPs within the cells via endocytosis; (**B**) enhanced cell death induced by GNPs; (**C**) generation of ROS and damage to cellular organelles (cytoskeleton and mitochondria) following 230 MeV proton beam irradiation [55]. Scale bar: 50 µm; ★: *p*-value < 0.02.

**Figure 7:**
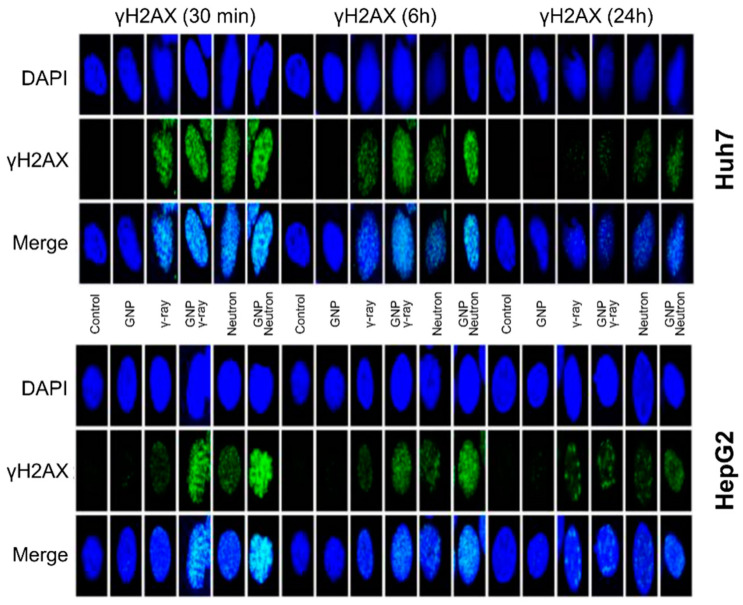
Radiation-induced DNA damage enhancement. Immunocytochemical staining of the DDR marker phosphorylated H2AX in Huh7 and HepG2 cells following γ-ray (5 Gy) and neutron radiation (5 GyE) exposure, with or without GNPs. Analysis conducted at 30 min, 6 h, and 24 h post-irradiation [119].

Numerous studies recently have employed different beams, such as proton, neutron, and ^12^C^6+^ ion beams, to probe RBE in various cell lines in the presence of (non)-functionalized GNPs (Table 3) [55,63,117,118,119,120,121]. Neutron radiation exhibits greater efficacy than low-LET radiation in treating certain cancer types, requiring only one-third of the equivalent photon dose for efficient cancer cell killing and enabling shorter treatment cycles [122,123,124]. Kim et al. discovered that utilizing GNP in conjunction with neutron particle therapy enhances cell viability in hepatocellular carcinoma cell lines [119]. Similarly, Lo et al. further demonstrated that GNPs, when combined with proton beam therapy, enhance the production of ROS, leading to increased damage to cellular organelles (cytoskeleton and mitochondria) and potentially improving the overall therapeutic effectiveness of proton therapy (Figure 6) [55]. These findings suggest that the synergy between GNPs and high-LET proton radiation could significantly enhance tumor cell cytotoxicity while sparing surrounding healthy tissues. Additionally, damage to DNA foci and sustained expression of phosphorylated H2AX were observed up to 24 h post-treatment in GNP-treated cells irradiated with either gamma or neutron radiation, with more significant damage seen after neutron exposure (Figure 7) [119].

## 3. Mechanisms of GNPs in Radiosensitization

**Figure 8:**
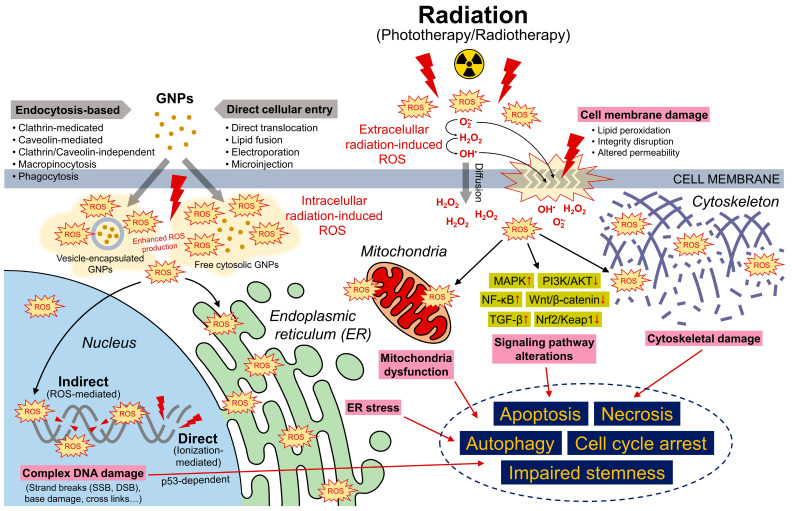
Schematic of ROS overproduction in the presence of GNPs under radiation, showing its effects on critical organelles and pathways, including DNA damage, ER stress, mitochondria dysfunction, and cytoskeletal instability, leading to apoptosis, cell cycle arrest, and functional loss.

ROS, crucial for cell processes such as proliferation, cell cycle regulation, motility and apoptosis, play a significant role in radiotherapy as they are generated during radiolysis, including O_2_^•−^, OH^•^, and H_2_O_2_, exerting toxicity towards both tumor cells and adjacent normal tissues [125,126]. Moreover, radiation can trigger the endogenous production of ROS in mitochondria, leading to alterations in mitochondrial membrane permeability and subsequently increasing ROS production [127]. Excessive levels of ROS disrupt mitochondrial electron transport, induce redox imbalances, and cause oxidative stress, while endogenous antioxidant systems, such as superoxide dismutases and catalase, protect against radiation-induced damage by scavenging free radicals [128,129,130]. In the ER, ROS production occurs through oxidoreductase Ero1 and NADPH oxidase (NOX). Ero1 plays a key role in oxidative protein folding, utilizing molecular oxygen to create disulfide bonds in newly folded proteins. The resulting luminal H_2_O_2_ from Ero1 and NOX is effectively neutralized by ER peroxidases, such as peroxiredoxin 4 (Prx4), GPx7, and GPx8, preventing H_2_O_2_ leakage from the ER. ROS-triggered unfolded protein response inactivates protein tyrosine phosphatase sulfhydration, promoting PKR-like endoplasmic reticulum kinase phosphorylation and regulating the balance between autophagy and apoptosis for cellular homeostasis (Figure 8) [131,132,133].

Previous studies have noted that when noble metal nanostructures are exposed to either continuous-wave or pulsed laser irradiation, ROS are generated, extending the scope beyond the utilization of GNPs as photothermal therapy agents [134,135]. Extensive experimental and theoretical investigations have been conducted to explore the potential of enhancing radiation therapy through the incorporation of GNPs. It has been observed in recent studies that during radiotherapy, GNPs can amplify the generation of ROS by causing deposited protons to collide with GNPs at the SOBP, inducing additional ejected electrons from high-Z GNPs [55,65,94,95,136]. Secondary electrons, photons (e.g., X-rays or γ emission), and positrons are induced from GNPs by high-energy protons with low LET, and these secondary electrons, upon interaction with water molecules, produce elevated levels of ROS [65]. Misawa and Takahashi demonstrated that the generation of ROS, such as radical OH^•^, O_2_^•−^, and ^1^O_2_, is enhanced by GNPs under X-ray irradiation, attributable to the emission of photo- and Auger-electrons as well as fluorescent X-rays produced in the interaction of incident X-rays with GNPs in the diagnostic range [58]. The enhancement of ROS formation by the protoporphyrin IX photosensitizer under light irradiation is also significantly pronounced in the presence of GNPs [137]. The intracellular localization of GNPs can vary significantly depending on factors such as size, shape, surface modifications, the method of cellular uptake, and the specific characteristics of the cell type. The uptake of spherical GNPs is primarily receptor-mediated, with optimal particle size (30–50 nm) and ligand density being important factors, although variations across cell types may exist [138]. Nonspherical particles typically exhibit more complex uptake behavior, with larger aspect ratios generally reducing effectiveness [139,140]. Surface functionalization with molecules such as PEG or molecular vectors can influence uptake, with PEGylation typically reducing efficiency and altering cellular entry mechanisms, while functionalization targeting tumor cell receptors can enhance delivery. Additionally, the surface charge of GNPs plays a pivotal role in uptake, with positively charged particles generally showing better internalization. The presence of serum proteins can alter GNP uptake, and the binding of proteins to nanoparticle surfaces can interfere with their interactions with cells [138].

Ionizing radiation from radiation therapy induces DNA damage through both ionization and the generation of ROS [4]. This damage manifests as base oxidation, single-strand breaks, and, notably, DBSs, with the latter being of utmost importance [141,142]. The occurrence of DBSs triggers the activation of key sensor proteins such as ataxia-telangiectasia-(mutated), Rad3-related, and DNA-dependent protein kinase within the DNA damage repair pathway. In response to these activation events, checkpoint kinases Chk1 and Chk2 undergo phosphorylation, activating the p53 protein during irradiation [143,144]. Subsequently, cells initiate repair mechanisms for DBSs, predominantly through either the non-homologous end joining (NHEJ) pathway or the homologous repair (HR) pathway. However, insufficient repair capabilities may lead to radiation-induced cell death [145,146].

Radical scavengers such as dimethyl sulfoxide (DMSO) have been employed in conjunction with GNPs and radiation [147]. The combination of GNPs, irradiation, and DMSO exhibited enhanced cell survival in comparison with samples treated solely with GNPs and irradiation, emphasizing DMSO’s effective hydroxyl radical scavenging without negatively impacting cell biology at a relevant concentration [61,148].

## 4. Promising Developments

GNP-based radiosensitization has experienced substantial growth due to interdisciplinary research involving radiobiology, physics, chemistry, and materials science. Although there have been notable advances in identifying novel nanoparticle formulations for preclinical investigations, the transition to clinical applications has encountered obstacles. There are currently two forms of GNPs, CYT-6091 and silica Core-Au shell nanoparticles (AuroShell^®^) that have been tested against certain types of cancer, with CYT-6091 being composed of PEGylated colloidal GNPs for delivering recombinant human tumor necrosis factor-alpha (rhTNF) against lung cancer, and AuroShell^®^ examined for photothermal ablation against head and neck carcinoma [149]. Beyond the use of GNPs as radiosensitizers, other metallic alternatives, such as bismuth, platinum, hafnium oxide, titanium dioxide, and magnetic nanoparticles, have been recently explored for multimodal cancer treatment. These materials enhance radiosensitization through various mechanisms, including radiation dose amplification, ROS overproduction, photothermal conversion, and immune modulation, offering promising strategies to improve radiotherapy efficacy [31,150,151,152,153,154]. The clinical assessment of GNPs for radiosensitizing purposes, however, remains unexplored, with a key challenge being the absence of in vivo evidence validating their radiotherapeutic efficacy. Various studies involving recombinant DNA and biological systems have demonstrated the potential of GNPs as radiosensitizers. Although significant progress has been achieved in laboratory settings, translating these findings to in vivo applications poses challenges. Many formulations lack colloidal stability, requiring intra-tumoral administration to mitigate particle aggregation and ensure sufficient gold concentration at tumor sites. Additionally, a significant challenge in the therapeutic use of GNPs is the potential for long-term toxicity arising from the prolonged presence of particles in the liver. Furthermore, unintended off-target effects may occur when GNPs interact with healthy tissues or organs that are not the primary targets of treatment, potentially leading to adverse reactions or reduced therapeutic efficacy. Albumin, a serum protein, for instance, can interact with GNPs in an unpredictable manner, potentially causing aggregation, which limits the effective dose of the particles at the tumor site and lowers their bioavailability [155,156,157]. Macrophages, part of the immune system, also recognize and engulf GNPs, which can lead to their premature clearance from circulation and reduce their accumulation at the intended target site [158]. To enhance the therapeutic efficacy of GNP radiosensitizers, it is essential to identify the factors influencing in vivo radiation amplification effects and incorporate these characteristics into formulation design.

## 5. Conclusions

The integration of radiation therapy with other therapeutic modalities, such as chemotherapy, stands as a promising strategy in the ongoing quest for more effective cancer treatments. Recognizing the limitations associated with radiation alone has prompted exploration into innovative approaches, and among these, nanoparticles have gained prominence. Noticeably, GNPs have emerged as an effective strategy to enhance the overall efficacy of cancer therapies. This is attributed, in part, to their ability to trigger the generation of ROS through secondary electrons produced by radiation exposure, enhancing the cytotoxic effects of cellar organelles (mitochondria, cytoskeleton, etc.). This pathway of ROS-induced cytotoxicity may act in conjunction with the DNA damage from radiotherapy for tumor treatment. As the exploration of nanotechnology in medicine progresses, the potential synergy between GNPs and radiotherapy opens up new horizons for personalized and targeted cancer treatments. Future research investigations in this direction hold the promise of refining our understanding of the intricate interplay between various treatment modalities, ultimately creating a new framework for more potent and tailored approaches to combat this complex and challenging disease. Recently, the use of GNPs to enhance the efficacy of proton therapy has been extensively studied [55,159,160,161]. This review may offer valuable insights for further research.

## Figures and Tables

**Table 1:** Radiosensitization of GNPs with X-ray irradiation.

Author	Particle Size (nm)	Conc.	Surface Coating	Cell Model	Source Energy	Radiosensitization Observation
Jain et al. [82]	1.9	12 μM	Thiol	MDA-MB-231	160 kVp	↓ Cell viability
Cui et al. [83]	2.7	0.5 μg/mL	Tiopronin	MDA-MB-231	225 kVp	↓ DNA repair
Khoshgard et al. [84]	47 52	50 μM	PEG Folate	HeLa	120–250 kVp	↑ Cell death
Chen et al. [85]	28	36 μg/mL	BSA	U87	160 kVp	↓ Clonogenicity ↑ DSBs ↑ Apoptosis ↓ Tumorigenicity
Soleymanifard et al. [86]	16	100 μM	Glucose	MCF7 QU-DB	100 kV 6 MV	↑ DNA damage ↓ Cell viability
Zhang et al. [87]	27.8	400 nM	Octaarginine	LS180	6 MV	↓ Clonogenicity ↑ Cell cycle arrest ↑ Apoptosis
Penninckx et al. [88]	10	50 μg/mL	Amino-PEG	A459 A431 MDA-MB-231 T98G PANC1	225 kV	↑ Cell death ↓ Thioredoxin reductase activity
Jia et al. [89]	2	2 µM	LNG	EC1	6 MV	↑ Apoptosis ↑ ROS generation ↓ Tumorigenicity
Bromma et al. [90]	20.9	0.2 nM	PEG/RGD	HeLa	6 MV	↓ Cell viability ↑ DSBs
Sürer et al. [91]	15	25 µg/mL	CTX CDDP CTX-CDDP	UPCI-SCC-131	6 MV	↓ Clonogenicity
Jing et al. [92]	20	100 µg/mL 150 µg/mL 200 µg/mL	Gallic acid	U251	6 MV	↓ Cell viability ↑ Cell cycle arrest ↑ Apoptosis
Mehrnia et al. [93]	10	12.5 µg/mL	AS1411	MCF-7 MDA-MB-231	4 MeV	↓ Cell viability
Hara et al. [94]	17.1	150 µg/mL 250 µg/mL	PSMA-targeted	LNCaP PC3 22Rv1	160 kVp	↓ Cell viability ↑ DBSs ↑ RIBE signaling ↓ Clonogenicity ↑ ROS generation
Jackson et al. [33]	11	7.5 µg/mL	PEG PEG/RGD	PC3 F12	6 MV	↓ DBSs ↓ Cell viability ↓ Tumor volume
Nosrati et al. [95]	13.7	40 μg/mL	Gd_2_O_3_@BSA	HUVEC 4T1	6 MV	↓ Cell viability ↓ Clonogenicity ↑ ROS generation ↓ Tumor volume
Khorshid et al. [96]	18.2	8 µM 16 µM	HApt-DSB	MCF-7 BT-474	6 MV	↓ Cell viability
Bemidinezhad et al. [97]	53.6	15 μg/mL	Glucose	B16F0	6 MV	↓ Clonogenicity ↑ Apoptosis ↑ ROS generation
Ghaffarlou et al. [98]	8.7	18.75–1500 μg/mL	Alg-DA	4T1	6 MV	↓ Cell viability ↓ Clonogenicity ↑ ROS generation

Conc., concentration; BSA, bovine serum albumin; DSB, double-strand break; LNG, levonorgestrel; CTX, cetuximab; CDDP, cisplatin; PSMA, prostate-specific membrane antigen; RIBE, radiation-induced bystander effect; RGD, integrin-binding domain RGD; Alg-DA, dopamine-conjugated alginate.

**Table 2:** Radiosensitization of GNPs with γ-ray irradiation.

Author	Particle Size (nm)	Conc.	Surface Coating	Cell Model	γ-Ray Source	Radiosensitization Observation
Zhang et al. [60]	4.8–12.1 27.3 46.6	50 μM 100 μM	PEG	HeLa	^137^Cs	↑ Apoptosis ↓ Tumor volume/weight
Kaur et al. [105]	5–9	5.5 μmol/mL	Glucose	HeLa	^60^Co	↑ Cell death
Zhang et al. [106]	<2	50 μg/mL	Glutathione BSA	HeLa	^137^Cs	↓ Tumor volume/weight ↑ DNA damage
Khoshgard et al. [84]	47 52	50 μM	PEG Folate	HeLa	^60^Co	↑ Cell death
Enferadi et al. [104]	<3	45 μg/mL 90 μg/mL	PEG-cRGDfK	ALTS1C1	^137^Cs	↓ Cell viability
Guo et al. [107]	14.4 30.5	25 μg/mL	PEG	H22 HepG2	^137^Cs	↓ Clonogenicity
Pérez-Amor et al. [108]	50	7 × 10^5^ np/mL	Citrate Transferrin	Caco2 SKBR3	^137^Cs	↑ γH2AX foci counts ↓ Cell viability
Shelar et al. [109]	16.5	60 μg/ml	Lanreotide peptide	CHO AR42J F-12K	^60^Co	↓ Cell viability ↓ Clonogenicity ↑ ROS generation ↑ Apoptosis

Conc., concentration; BSA, bovine serum albumin; cRGDfK, cyclic-(arginine-glycine-aspartic acid-phenylalanine-lysine); np, nanoparticles.

**Table 3:** Radiosensitization of GNPs with particle radiation.

Author	Particle Size (nm)	Conc.	Surface Coating	Cell Model	Particle Beam	Radiosensitization Observation
Kaur et al. [118]	5–9	1.96–29.2 μM	Glucose	HeLa	^12^C^6+^	↓ Cell viability
Li et al. [117]	5 10	50 μg/mL	NF	A431	Proton	↓ Cell viability ↑ ROS generation
Kim et al. [119]	5	1 mM	NF	HepG2 Huh7	Neutron	↓ Cell viability ↑ Apoptosis ↑ Cell cycle arrest ↑ DNA damage ↓ Cell migration ↓ Cell invasion
Li et al. [63]	3–5	5 μg/mL	Cetuximab	A431	Proton	↓ Cell viability ↑ ROS generation
Cunningham et al. [120]	50	10 μg/mL	NF	CHO-K1	Proton	↓ Cell viability ↑ DNA damage
Zhang et al. [121]	15.97	6 μg/mL	11-MUA	B16-F10	^12^C^6+^	↓ Cell viability ↓ Tumor growth rate ↑ Necrosis ↑ Apoptosis
Lo et al. [55]	55	80 μg/mL	NF	A431	Proton	↓ Cell viability ↑ ROS generation ↑ Cytoskeleton damage ↑ Mitochondria damage

Conc., concentration; NF, non-functionalized; HBT, human brain tissue; 11-MUA, 11-mercaptoundecanoic acid.
